# Imaging of supratentorial ependymomas with radio-pathological correlation

**DOI:** 10.37349/etat.2024.00245

**Published:** 2024-06-27

**Authors:** Arpita Sahu, Aditi Venkatesh, Aman Snehil, Abhishek Mahajan, Amit Janu, Ayushi Sahay, Epari Sridhar

**Affiliations:** National Technical University of Athens (NTUA), Greece; ^1^Department of Radiodiagnosis, Tata Memorial Hospital (Homi Bhabha National Institute), Mumbai, Maharashtra 400012, India; ^2^Department of Pathology, Tata Memorial Hospital (Homi Bhabha National Institute), Mumbai, Maharashtra 400012, India; ^3^Department of Imaging, The Clatterbridge Cancer Centre NHS Foundation Trust, L7 8YA Liverpool, UK; ^4^Faculty of Health and Life Sciences, University of Liverpool, L7 8TX Liverpool, UK

**Keywords:** Supratentorial, ependymoma, ZFTA, radiopathological correlation, stellate sign

## Abstract

**Aim:**

Supratentorial ependymoma (STE) is a rare tumor with distinct genetic alterations, whose imaging features have been scarcely studied. This study aims to review the computed tomography (CT) and magnetic resonance imaging (MRI) features of a cohort of histopathologically proven STE to identify the distinguishing features of STE, and look for specific signs of zinc finger translocation associated (ZFTA) fused STEs.

**Methods:**

Ethical clearance was obtained from the institutional ethics committee. The magnetic resonance (MR) images, CT images when available, clinical details, and pathological reports of 25 patients from a single institute with histopathologically proven STE were retrospectively reviewed. Imaging features, demographic details, pathological and molecular features, and type of surgical resection were described and tabulated. Relevant associations with imaging features were computed and tabulated.

**Results:**

The study showed that STEs are common in the pediatric population with no sex predilection. The periventricular location was the most common. A significant association between periventricular location and the presence of a cystic component (*P* value = 0.023) and the presence of the periwinkle sign/stellate sign (*P* value = 0.045) was found. Common features of ZFTA fused STEs included periventricular or intraventricular location, cystic component, necrosis, and the periwinkle sign. A significant association was found between ZFTA fusion and cystic component (*P* value = 0.048).

**Conclusions:**

This study attempts to identify the imaging features of STEs and their associations with molecular pathology and surgical outcome, and the distinguishing features of ZFTA fused STEs.

## Introduction

Ependymomas are tumors arising from the ependymal cells of the ventricles, central canal, Virchow-Robin spaces, and cortical rests [1, 2] that account for about 3–5% of all adult intracranial gliomas [3, 4]. They also arise from the cells lining the central canal in the spinal cord [5–8] and are a heterogeneous group of tumors with distinct genetic alterations in each location. In a minority of cases, ependymomas arise from the supratentorial parenchyma and show no continuity with the ventricular system. These ependymoma variants are called ectopic, cortical, lobar, or extra-ventricular ependymomas, and these are rare compared to the infratentorial counterpart. Supratentorial ependymomas (STEs) account for nearly one-third of all intracranial ependymomas, and 42% of STEs occur in children [9]. A unique phenomenon that has been noted is the presence of STEs distant from the ventricular system within the actual cerebral parenchyma [6].

About 45–65% of STEs have been appreciated in this extra ventricular site, and a correlation between distance of the neoplasm from the midline cerebral structures and worsening grade has also been suggested [10–12]. Additional data indicate that patients with hemispheric ependymomas have decreased progression-free survival (PFS) and overall survival (OS) compared with tumors occurring in the third or lateral ventricles [13, 14]. Although often considered an intraventricular tumor, more than half of STEs occur within the cerebral hemispheres [4, 13, 15, 16]. Their hemispheric location likens ependymomas to the other primary intra-axial gliomas.

In adults, the majority of supratentorial lesions are classified as World Health Organization (WHO) grade 3 [10, 12]. Evidence supports the idea that STEs have a worse prognosis than infratentorial tumors in adults [13, 14, 17–21]. According to the latest WHO 2021 classification, they are classified as: zinc finger translocation associated (ZFTA) fusion positive, and yes-associated protein 1 (YAP1) fusion positive [22]. ZFTA is the current term used for *C11orf95* gene, which is said to be more representative of this type of ependymoma than v-rel reticuloendotheliosis viral oncogene homolog A (RELA), as ZFTA may fuse with genes other than RELA [22]. ZFTA fused ependymomas have been found to have a poorer prognosis than other molecular subtypes [17, 22].

There has been no consensus within the neuro-oncologic community regarding the best treatment for adult patients with STEs and their anaplastic counterparts [23, 24]. Some centres treat adults with STEs like their pediatric counterparts, and other institutions treat them as patients with any other supratentorial glioma. The location of STEs has been shown to affect the surgical outcome, with parenchymal tumors having a greater percentage of gross total resection (GTR), while those closer to the ventricles have a higher chance of near total or sub-total resection (STR) [24]. This study aims to describe the computed tomography (CT) and magnetic resonance imaging (MRI) features of a cohort of histologically proven STEs and identify the specific features of ZFTA fused STEs.

## Materials and methods

This was a retrospective study where the magnetic resonance (MR) images, CT images when available, clinical details, and pathological reports of 25 patients from a single institute with histopathologically proven STEs presenting at a tertiary cancer institute between July 2009 to November 2021 were reviewed. Recurrent tumors were excluded from the study. Ethics approval was obtained after evaluation by the institutional review board and waiver of consent was obtained.

### Clinical data

A total of 25 patients met the inclusion criteria. The mean age at diagnosis was 13 years (range 2–45 years). The number of male patients was 12, and 13 patients were female.

### Imaging analysis

All patients were examined with brain MRI at 1.5 T (Philips Ingenia) scanner. The MRI sequences were performed were: T2 fast spin-echo [time of repetition (TR)/time of echo (TE), 2,700/100), fluid-attenuated inversion recovery (FLAIR: TR/TE, 9,000/120; inversion time, 2,200 ms), unenhanced T1 spin-echo and at least two planes of contrast-enhanced T1 spin-echo [TR (range)/TE, 600–700/20]. Axial susceptibility-weighted images (TR/TE, 570/30 at 1.5 T) and axial diffusion-weighted images (TR/TE, 8,300/70 at 1.5 T; b 1,000 s/mm^2^, 4 mm thickness).

Various MRI features were evaluated for every case. CT images were reviewed wherever available. All images were independently reviewed by a single neuroradiologist with 12 years of experience in neuroimaging.

MRI assessments included: the location of the mass, presence of solid, and cystic components, their signal intensities on T1- T2-weighted (T1W, T2W), FLAIR, and susceptibility weighted imaging (SWI) images, restricted diffusion within the solid component, enhancement characteristics, presence of calcification, and presence or absence of the “periwinkle sign” or “stellate sign”.

The solid component was characterized as showing high intensity, low intensity, or isointensity relative to grey matter if most (> 90%) of the solid portion had homogeneous signal intensity on T2W images, and heterogeneous if the solid component revealed varying intensities on T2W images. The enhancement pattern of the solid component was defined as homogeneous if enhancement was present in the whole of the tumor volume and heterogeneous, if ring enhancing areas with an irregular rim suggestive of necrosis, were noted within the solid component.

The characteristics of the cystic component assessed were the size of the cyst, signal intensity on T2 and FLAIR relative to cerebrospinal fluid (CSF), presence/absence of blood within the cyst, and presence/absence of wall enhancement.

### Pathological analysis

All the cases were histopathologically confirmed. Histological grade and Mindbomb homolog-1 (Mib) labelling index were noted.

Molecular pathology: testing for ZFTA fusion was available for 8 (32%) of cases. ZFTA fusion was considered positive if L1 cell adhesion molecule (L1CAM) overexpression was positive. Testing for CyclinD1 overexpression was available in 12 cases. Testing for L1CAM and CyclinD1 overexpression was done using immunohistochemistry.

Testing for p53 overexpression was done using immunohistochemistry and was available in 18 cases. Testing for YAP1 was not available.

### Surgical outcome

GTR was considered when no residual tumor was identified on postoperative MRI. Near total resection (NTR) was when > 90% of the tumor was resected and some tumor tissue was left behind. STR was defined as resection of 50–90% of the tumor.

### Statistical analysis

Imaging features including size, location, signal intensity, enhancement, diffusion restriction, and presence of calcification and hemorrhage were described and tabulated. Demographic details, pathological and molecular features, and type of surgical resection were also described, and relevant associations with imaging features were computed and tabulated. All statistical analysis was performed using SPSS software (IBM Corp), version 20.2. *P* value for categorical variables was calculated using the Chi-square test or Fisher exact test, and for continuous variables was calculated using the Mann-Whitney U test since data is not normally distributed.

## Results

### Demographic features

Of all the 25 cases in our study, most cases were in the pediatric age group (≤ 18 years of age), with the mean age being 13 years (range 2–45 years). Three patients were below 5 years of age, and 8 patients were teenagers (between 13–19 years of age). Thirteen patients were between 5–12 years of age. A similar age predilection has been documented by other studies [24, 25]. Three patients were in the adult age group. The mean age among ZFTA fused cases was 7 years (range 2–12 years). No sex predilection was observed.

### Imaging features

Location: 15 (60%) cases were in the periventricular location, 4 (16%) cases were intraventricular, and 6 (24%) cases were purely cortical. Eleven (44%) of cases were situated in the frontal lobe. A predilection for the left side with 16 (64%) cases was noted.

**Figure 1:**
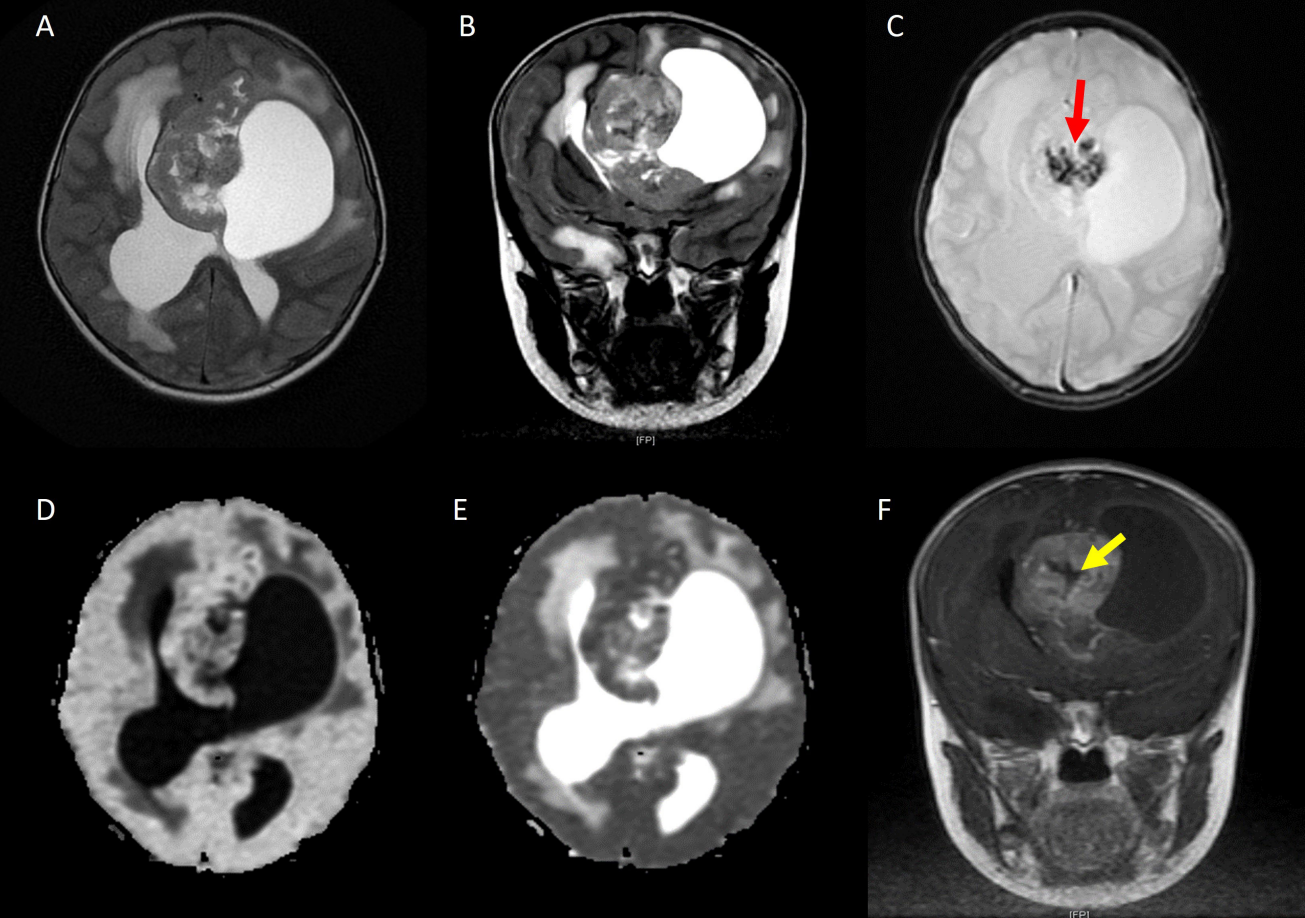
STE in periventricular location. A. Axial T2W image and B. coronal T2W image showing a heterogeneous solid cystic mass in the periventricular region; C. axial SWI showing foci of blooming (red arrow) suggestive of calcification/hemorrhage; D. diffusion-weighted imaging (DWI) shows the tumor is bright; E. apparent diffusion coefficient (ADC) shows hypointense signal suggesting low ADC value; F. coronal contrast-enhanced T1W image shows heterogeneous post contrast enhancement with the central non-enhancing area (yellow arrow) suggestive of necrosis

The typical appearance of STE in the periventricular location is demonstrated in Figure 1.

**Figure 2:**
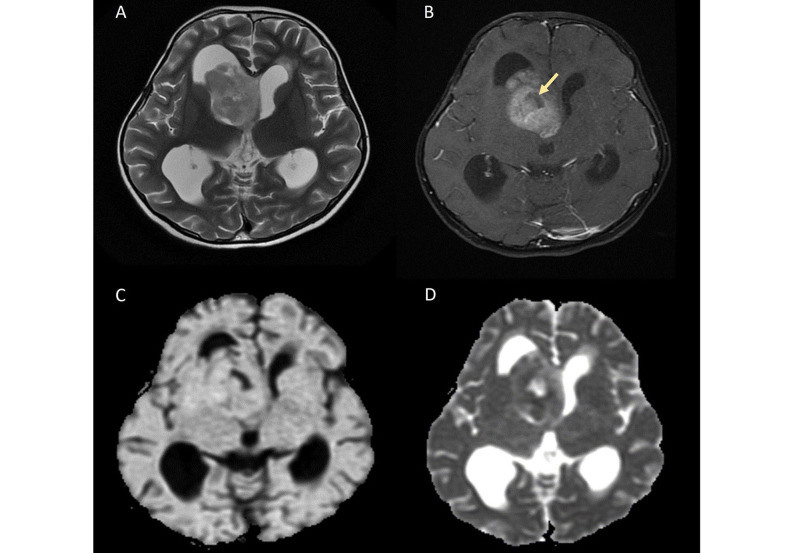
Intraventricular STE. A. Axial T2W image shows a heterogeneously hyperintense solid mass arising from the right lateral ventricle; B. axial contrast-enhanced image demonstrates heterogeneous enhancement of the mass with areas of necrosis (yellow arrow); C. on diffusion-weighted imaging (DWI) and D. apparent diffusion coefficient (ADC) map, the lesion shows patchy areas of restricted diffusion

Intraventricular STE is depicted in Figure 2.

**Figure 3:**
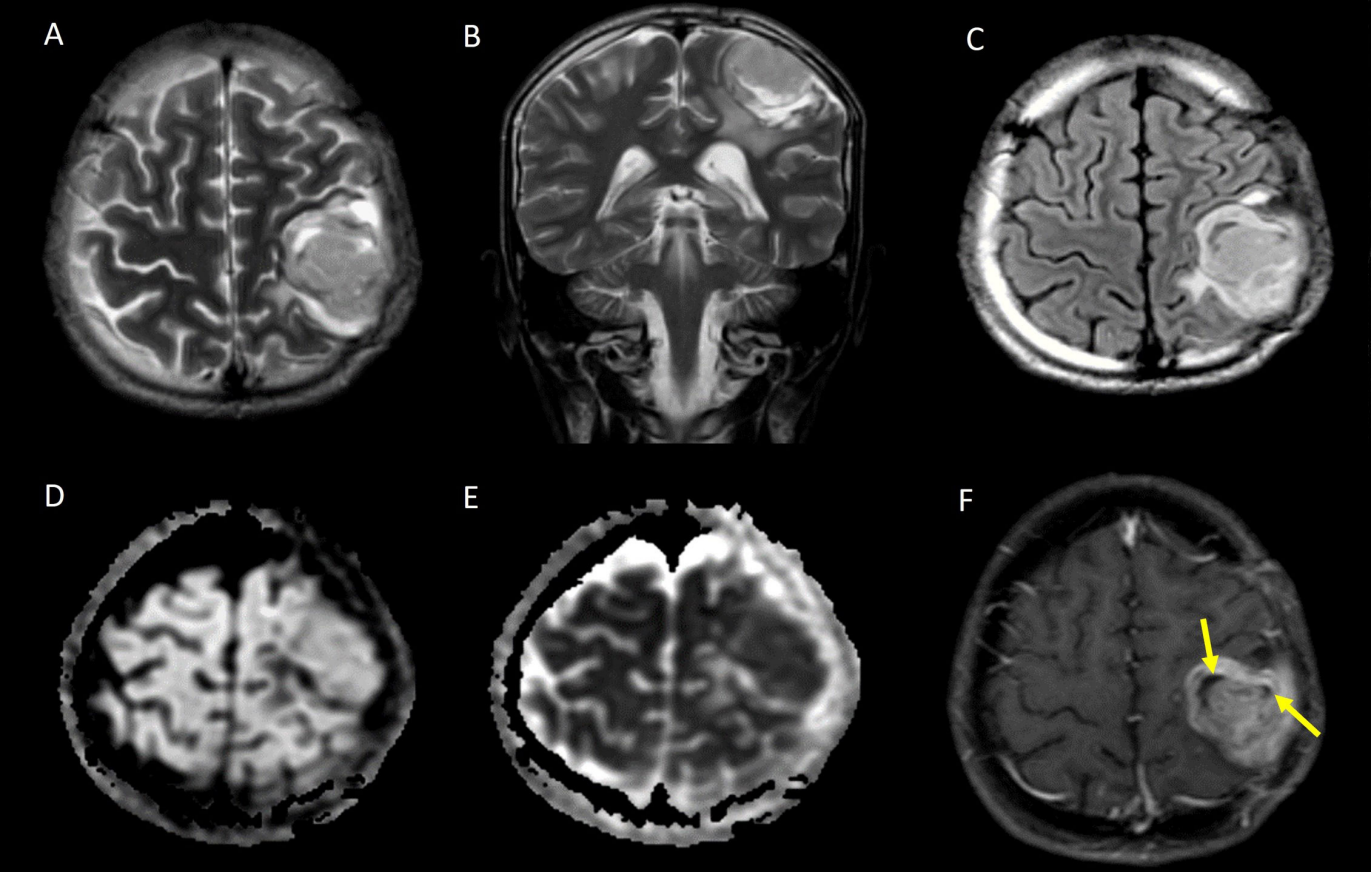
Cortical based STE. A. Axial T2W image and B. coronal T2W image showing a heterogeneously hyperintense solid mass with few cystic areas in the cortex of the left frontal lobe; C. axial T2 FLAIR image shows the cystic areas are not suppressed; D. the lesion appears isointense on diffusion-weighted imaging (DWI); E. it shows low apparent diffusion coefficient (ADC) values suggestive of restricted diffusion; F. axial contrast-enhanced T1W image showing heterogeneous post contrast enhancement, with the cystic areas corresponding to non-enhancing areas of necrosis (yellow arrows)

The cortical location of STE is depicted in Figure 3.

Size: the mean maximum axial dimension was 6.5 cm (range 1.5–11 cm). The mean maximum axial dimension for the periventricular location was 8 cm (range 5.5–10.9 cm), for the intraventricular location was 5 cm (range 1–9 cm), and for the cortical location was 4.5 cm (range 2.6–6.5 cm). The mean size of the cystic component in terms of the maximum axial dimension was 4.1 cm and the largest axial dimension of the solid component was 4.6 cm.

Morphology: 19 (76%) cases had a solid and cystic component. The cystic component was hyperintense to CSF on FLAIR, as also observed by Nowak et al. [17], with blood within the cyst in 5 (26%) of cases. The wall of the cystic component showed post-contrast enhancement in 89.5% (*n* = 17) cases.

The solid component was well-defined, lobulated, and had variable appearances on T1 and T2 weighted sequences, and all cases showed heterogeneous enhancement in the solid component on post contrast sequences and 20 (80%) of cases showed areas of necrosis.

**Figure 4:**
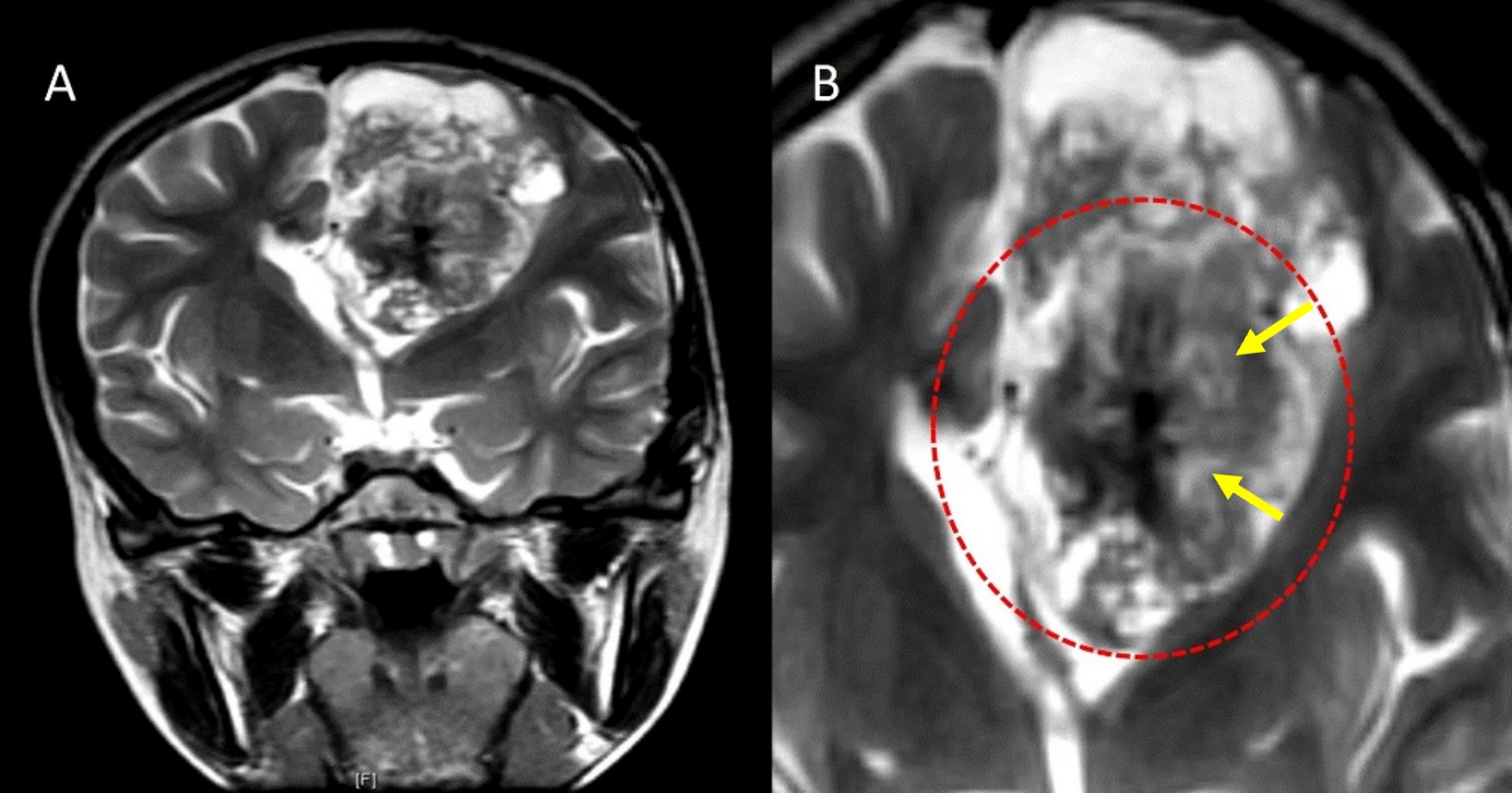
Periwinkle sign. A. Coronal T2W image and B. close-up image of the tumor on coronal T2W image showing a large heterogeneously hyperintense solid cystic lesion in the left frontal lobe in the periventricular location with central T2 hypointense area with calcification and central necrosis (yellow arrows), resembling the shape of a periwinkle flower (red dotted circle)

**Figure 5:**
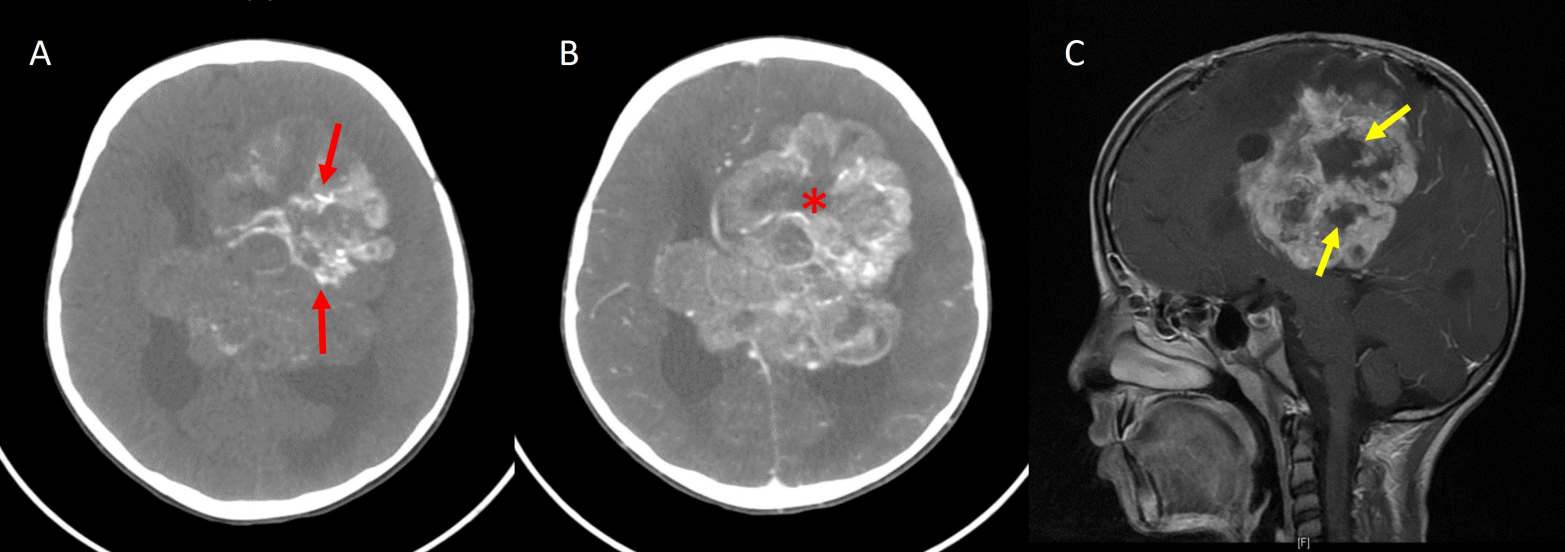
Periwinkle sign. A. Axial noncontrast-enhanced CT image shows a large heterogeneous mass in the periventricular location of the left frontoparietal lobe with centripetal calcification (red arrows); B. axial contrast-enhanced CT image showing heterogeneous enhancement of the lesion with the central necrotic area (asterisk), resembling the shape of a flower; C. sagittal contrast-enhanced T1W image of the same patient also shows a central non-enhancing area of necrosis in the shape of a flower (yellow arrows)

**Figure 6:**
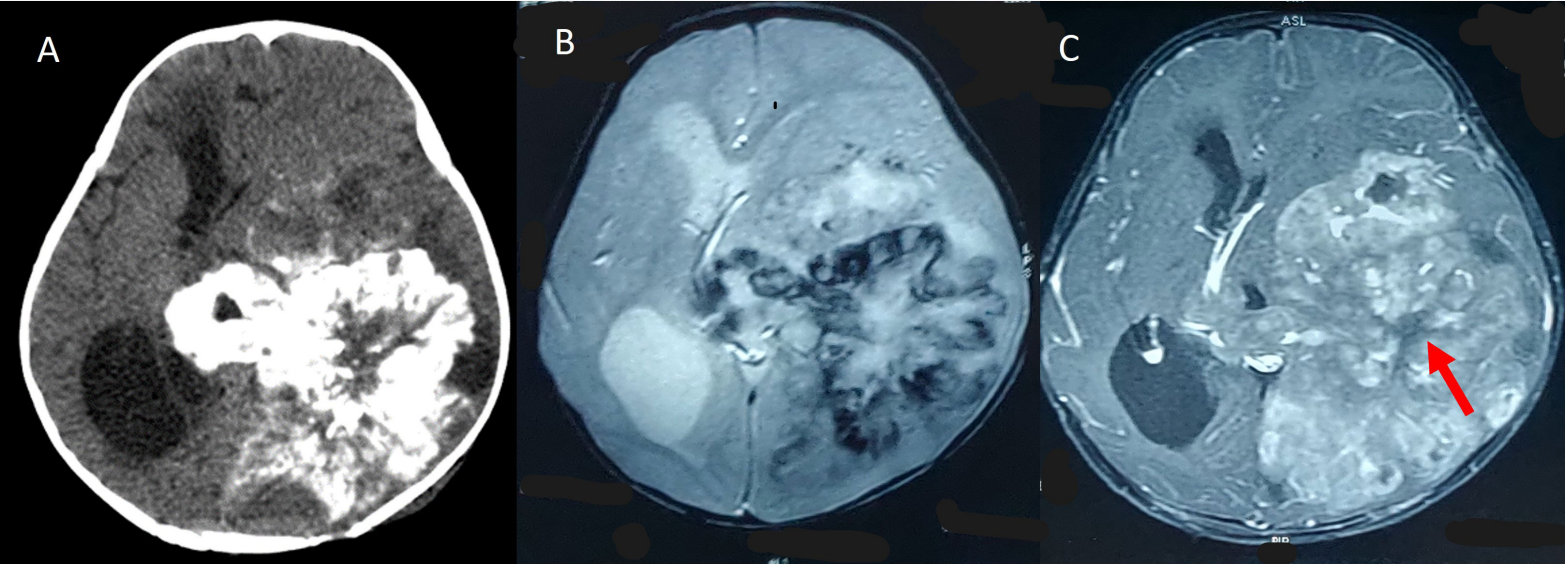
Periwinkle sign. A. Axial noncontrast-enhanced CT image showing a large heterogeneous mass in the left temporoparietal lobe in the periventricular location, with dense centripetal calcification giving rise to a flower like shape; B. axial SWI shows a similarly shaped area of blooming; C. contrast-enhanced T1W MR image shows a central non-enhancing area of necrosis (red arrow)

Blooming was seen in 20 cases on susceptibility-weighted MR imaging, out of which 13 cases had CT imaging, and all showed calcification on CT. The “periwinkle sign”, i.e. the flower-like appearance of the tumor with the central area of necrosis and centripetal calcification [25], was observed in 17 (68%) cases. Another pattern of calcification resembling a spoke wheel was observed in these cases, which has been termed the “stellate sign”. The periwinkle sign on MRI and CT is demonstrated in Figures 4, 5, and 6. A significant association was found between the periwinkle/stellate sign and the periventricular location (*n* = 13, 86.7%, *P* value = 0.045). It was observed less frequently in the intraventricular location (*n* = 2, 50%), and purely cortical location (*n* = 1, 33%).

The demographic and imaging features are summed up in Table 1.

**Table 1:** Demographic and imaging features

**Variable**	**Number of cases (%)**		
Age	Mean (SD)	13	
Gender	Female	13 (52.0)	
	Male	12 (48.0)	
Laterality	Left	16 (64.0)	
	Right	9 (36.0)	
Lobe	Frontoparietal	7 (28.0)	
	Frontal		11 (44.0)
	Frontotemporal		2 (8.0)
	Parietooccipital		3 (12.0)
	Parietotemporal		2 (8.0)
Size	Mean (SD)		6.5
Location	Intraventricular		4 (16.0)
	Purely cortical		6 (24.0)
	Periventricular		15 (60.0)
T1 signal	Hyperintense		7 (28.0)
	Isointense		18 (72.0)
Hemorrhage	No		17 (68.0)
	Yes		8 (32.0)
T2 signal	Hyperintense		1 (4.0)
	Hypointense		11 (44.0)
	Isointense		13 (52.0)
Postcontrast T1	Heterogeneous		25 (100.0)
Restricted diffusion	No		3 (12.0)
	Yes		22 (88.0)
SWI hypointensity	No		5 (20.0)
	Yes		20 (80.0)
Cystic component	Yes		19 (76.0)
	No		6 (24.0)
Cyst T1 signal	Hyperintense		18 (94.7)
	Hypointense		1 (5.3)
Cystic post contrast enhancement	No		2 (10.5)
	Yes		17 (89.5)
Blood in cyst	No		14 (73.7)
	Yes		5 (26.3)
Cyst size	Mean (SD)		4.1
Cyst FLAIR signal	Hyperintense		18 (72.0)
	Hypointense		1 (4.0)
	NAD		6 (24.0)
Necrosis	No		5 (20.0)
	Yes		20 (80.0)
Solid component size with necrosis	Mean (SD)		4.6
Edema	No		6 (24.0)
	Yes		19 (76.0)
Periwinkle sign/Stellate sign	No		8 (32.0)
	Yes		17 (68.0)
Calcification on CT	No		0 (0.0)
	Yes		13 (100.0)

SD: standard deviation

### Grade and molecular pathology

The number of Grade 3 cases was 23 (92%) and 2 (8%) cases were grade 2 tumors. The mean Mib labelling index was 9–11%.

**Figure 7:**
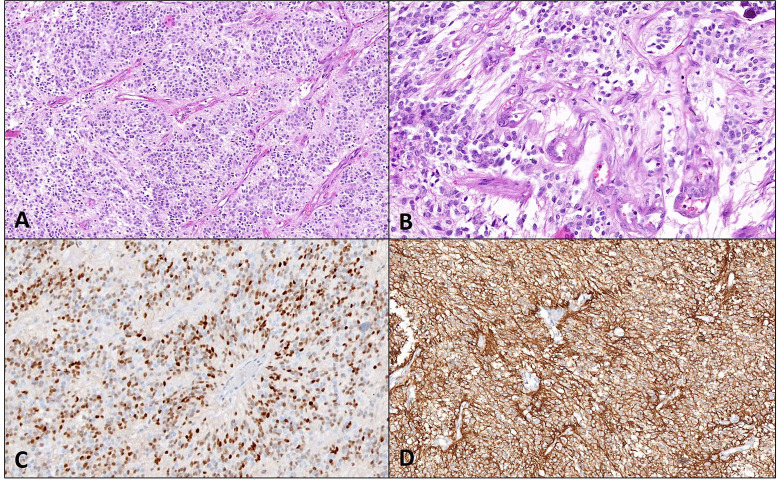
Pathological and molecular features of STE. A case of STE with A. perivascular arrangement (40x magnification) and B. microvascular proliferation (400x magnification) on histopathology. C. Diffuse positivity for CylinD1 and D. L1CAM is noted on immunohistochemistry (200x magnification each)

Cases were considered ZFTA fusion positive if L1CAM overexpression was positive. Testing for ZFTA fusion was available for 8 (32%) of cases, out of which 5 (62.5%) cases were ZFTA fusion positive and 3 (37.5%) of cases were negative. Testing for CyclinD1 overexpression was available in 12 cases, out of which 11 were positive and 1 was negative. It is associated with poor prognosis [17]. Histopathology and immunohistochemistry results in a ZFTA fusion positive case are shown in Figure 7.

**Figure 8:**
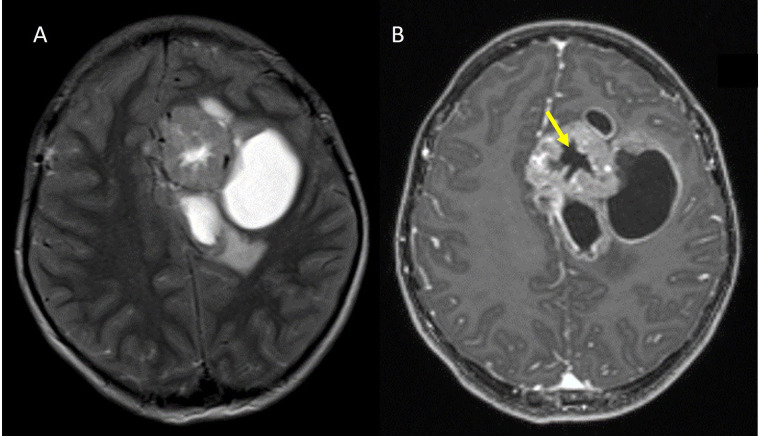
Stellate sign in a ZFTA fused case. A. Axial T2W image in a ZFTA fusion positive case shows a heterogeneously hyperintense solid cystic mass in the left frontal lobe in the periventricular location; B. axial contrast-enhanced T1W image shows a stellate central non-enhancing area of necrosis (yellow arrow)

Of the ZFTA fusion positive cases, 60% (*n* = 3) were periventricular in location, and 40% (*n* = 2) were intraventricular in location. None were purely cortical. All 5 ZFTA fused cases were associated with a cystic component, had necrosis, and showed the periwinkle/stellate sign. Figure 8 depicts the Stellate sign in a ZFTA fused case. A significant correlation was found between the presence of ZFTA fusion and the presence of cysts (*P* value = 0.048). All 5 ZFTA fused cases in this study were WHO grade 3.

Testing for p53 overexpression was available in 18 cases, out of which 12 (66.7%) were positive and 6 (33.3%) were negative. A significant association was found between the presence of p53 overexpression and necrosis (*P* value = 0.005) with 100% (*n* = 12) of p53 positive cases showing necrosis, and two p53 negative cases showing necrosis.

The pathological features are summed up in Table 2.

**Table 2:** Pathological and molecular features

**Pathological/Molecular feature**	**Number of cases (%)**	
Histopathological Grade	Grade 2	2 (8.0)
	Grade 3	23 (92.0)
CyclinD1	Negative	1(8.3)
	Positive	11 (91.7)
Mib labelling Index	Mean (SD)	11.4 (9.1)
ZFTA fusion (L1CAM overexpression)	Negative	3 (37.5)
	Positive	5 (62.5)
p53	Negative	6 (33.3)
	Positive	12 (66.7)

The associations of ZFTA fusion with imaging features are summed up in Table 3.

**Table 3:** Associations of ZFTA fusion with imaging features

**ZFTA fusion**	**Location**			**Cyst**	**Periwinkle/Stellate sign**	**Necrosis**
	**Intraventricular**	**Periventricular**	**Purely cortical**			
Positive	2	3	0	0	5	5
Negative	2	1	0	5	1	1
*P* value	> 0.999	> 0.999	0.167	0.048	0.238	0.238

### Surgical outcome

A total of 64% (*n* = 16) of cases underwent GTR, 24% (*n* = 6) cases underwent NTR and 12% (*n* = 3) of cases underwent STR. It was observed that smaller lesions were found to be more likely to undergo GTR, and larger lesions underwent NTR or STR. Purely cortical lesions were found to have higher rates of GTR, with all 6 of the cortical lesions observed undergoing GTR, while 53% (*n* = 8) and 50% (*n* = 2) of periventricular and intraventricular lesions respectively underwent GTR. ZFTA fused cases had higher rates of NTR (60%, *n* = 3), with 20% (*n* = 1) of cases undergoing STR and the remaining 20% (*n* = 1) of cases undergoing GTR.

There was a significant association between the periventricular location of the tumor with presence of a cystic component, (*P* value = 0.023) with 93% (*n* = 14) of periventricular tumors in this study showing a cystic component. 50% (*n* = 2) of intraventricular tumors also showed a cystic component, and 50% (*n* = 3) of purely cortical tumors had a cystic component.

The imaging features and associations according to intraventricular, cortical, and periventricular locations are summed up in Table 4.

**Table 4:** Associations of imaging features according to location

**Location**		**Intraventricular**		**Purely Cortical**		**Periventricular**	
		**Total (%)** ***n* = 4**	***P* value**	**Total (%)** ***n* = 6**	***P* value**	**Total (%)** ***n* = 15**	***P* value**
Cyst	No	2 (50.0)	> 0.999	3 (50.0)	0.125	1 (6.7)	0.023
	Yes	2 (50.0)		3 (50.0)		14 (93.3)	
Resection	GTR	2 (50.0)	0.38	6 (100)	0.109	8 (53.3)	0.353
	NTR	1 (25.0)		0 (0.0)		5 (33.3)	
	STR	1 (25.0)		0 (0.0)		2 (13.3)	
p53	Negative	1 (25.0)	> 0.999	1 (33.3)	> 0.999	4 (36.3)	0.638
	Positive	3 (75.0)		2 (66.7)		7 (63.3)	
Periwinkle sign/Stellate sign	No	2 (50.0)	> 0.999	4 (66.7)	0.059	2(13.3)	0.045
	Yes	2 (50.0)		2 (33.3)		13 (86.7)	

## Discussion

The aim of this study was to examine the imaging features of STEs, correlate these features with molecular and histopathological features, and examine their effect on surgical outcome. This study is the largest single institutional study of MR imaging features of STEs.

Based on their location, STEs have been described as intraventricular, intraparenchymal, and purely cortical [26]. They are more commonly parenchymal in location than intraventricular [25]. The results of this study are in accordance with this, with 15 (60%) cases in the periventricular location. Purely cortical lesions were found to have higher rates of GTR, as also documented by Metellus et al. [24]. The frontal lobe was the most frequent site of the tumor.

Imaging features of STEs observed include large size, heterogeneous appearance on T1 and T2 with areas of hemorrhage and necrosis, and solid and cystic component. A significant association was found between periventricular location of the tumor with presence of cystic component, (*P* value = 0.023) with 93% (*n* = 14) of periventricular tumors in the study showing a cystic component, while was observed less frequently in intraventricular and cortical tumors.

Calcification is a frequently observed feature [8, 11, 25–28], and 80% of cases showed SWI hypointensity. The periwinkle sign [25] or stellate sign, i.e. the flower/star-shaped configuration of central necrosis and centripetal calcification, was observed in 17 (64%) of cases, with a significant correlation between the presence of this sign and the periventricular location of the tumor (*P* value = 0.045). This sign can be helpful in identifying STEs and differentiating them from other tumors. On imaging, the differentials for STE would include atypical teratoid rhabdoid tumor (ATRT), embryonal tumor with multilayered rosettes (ETMR), and high-grade gliomas as these may have overlapping features with STEs, including large size, cystic/necrotic areas, and calcification [29, 30]. Age can help prioritize the order of differential diagnoses in such cases, although it remains difficult to rule out the other differentials.

Histological grade has been found to be an important prognostic factor for supratentorial STEs [24]. Worsening grade with increased distance of the tumor from the ventricles has also been reported [31]. A total of 23 (92%) of cases were grade 3, and 2 (8%) of cases were grade 2 tumors. The current sample size does not have adequate representation of grade 2 tumors for us to determine the association of the tumor grade and location or surgical outcome with adequate statistical significance.

ZFTA fused STEs have been identified by the WHO as a separate clinicopathological entity and have been found to have a poorer prognosis than other molecular subtypes [17, 22]. Genetic testing may not always be available so it is of interest to examine robust imaging biomarkers which may serve as surrogates to molecular testing and allow differentiation of this subset of ependymomas. Common imaging features of ZFTA fused ependymomas in this study included the periventricular location, the presence of cystic component (*P* value = 0.048), the Periwinkle/stellate sign, and necrosis. Other studies have reported cortical location more commonly in ZFTA fused STE, with intraventricular location being rare [17]. The presence of cyst, necrosis, and calcification in ZFTA fused STEs was also reported by Nowak et al. [17].

A significant association was found between the presence of p53 mutation and necrosis (*P* value = 0.005) with 100% (*n* = 12) of p53 mutated cases showing necrosis.

In summary, periventricular location, solid and cystic components, the presence of calcification and necrosis, and the periwinkle/stellate sign are distinctive features that differentiate STEs from other tumors and can aid in their diagnosis. The presence of a cystic component, necrosis, and the periwinkle sign were common features found in the ZFTA fused STEs in the study. Although this study is one of the few to examine the imaging features of STEs and attempt to describe the MRI phenotype of ZFTA fused STEs, it is limited by a relatively small sample size and molecular testing not being available in all patients. Further studies with a larger sample size and prospective design are warranted to identify these tumors and their molecular subtypes on imaging.

## Abbreviations

CT: computed tomography
FLAIR: fluid-attenuated inversion recovery
GTR: gross total resection
L1CAM: L1 cell adhesion molecule
MR: magnetic resonance
MRI: magnetic resonance imaging
NTR: near total resection
STE: supratentorial ependymoma
STR: sub-total resection
SWI: susceptibility weighted imaging
T1W: T1 weighted
TE: time of echo
TR: time of repetition
WHO: World Health Organization
ZFTA: zinc finger translocation associated

## Declarations

### Availability of data and materials

The datasets for this manuscript are not publicly available because data contains personally identifiable information that may be restricted from public access to protect individuals’ privacy.

### Copyright

© The Author(s) 2024.
